# Bacterial survival in radiopharmaceutical solutions: a critical impact on current practices

**DOI:** 10.1186/s41181-023-00221-3

**Published:** 2023-10-26

**Authors:** Julien Leenhardt, Luc Choisnard, Maelle Plasse, Valérie Ardisson, Nicolas de Leiris, Loic Djaileb, Pierrick Bedouch, Marie-Dominique Brunet

**Affiliations:** 1 Univ. Grenoble Alpes, INSERM, CHU Grenoble Alpes, LRB, 38000 Grenoble, France; 2 Univ. Grenoble Alpes, Domaine Universitaire de Grenoble, DPM, UMR CNRS 5063, 470, rue de la Chimie, 38400 Saint Martin d’Hères, France; 3 Univ. Grenoble Alpes, CHU Grenoble Alpes, Pharmacy , 38000 Grenoble, France; 4 Cancer Institute Eugène Marquis, Department of Nuclear Medicine, 35000 Rennes, France; 5 Univ. Grenoble Alpes, CNRS 5525, TIMC-IMAG, Grenoble, France

## Abstract

**Background:**

The aim of this brief communication is to highlight the potential bacteriological risk linked to the processes control of radiopharmaceutical preparations made in a radiopharmacy laboratory. Survival rate of *Pseudomonas aeruginosa* (*ATCC: 27853*) or *Staphylococcus aureus (ATCC: 25923)* or *Staphylococcus epidermidis (ATCC: 1228)* in multidose technetium-99 m solution was studied.

**Results:**

Depending on the nature and level of contamination by pathogenic bacteria, the lethal effect of radioactivity is not systematically observed. We found that *P. aeruginosa* was indeed affected by radioactivity. However, this was not the case for *S. epidermidis*, as the quantity of bacteria found in both solutions (radioactive and non-radioactive) was rapidly reduced, probably due to a lack of nutrients. Finally, the example of *S. aureus* is an intermediate case where we observed that high radioactivity affected the bacteria, as did the absence of nutrients in the reaction medium. The results were discussed in the light of current practices on the sterility test method, which recommends waiting for radioactivity to decay before carrying out the sterility test.

**Conclusion:**

In terms of patient safety, the results run counter to current practice and the latest EANM recommendation of 2021 that radiopharmaceutical preparations should be decayed before sterility testing.

**Supplementary Information:**

The online version contains supplementary material available at 10.1186/s41181-023-00221-3.

## Introduction

Radiopharmaceuticals are medicines used for diagnosis or therapy in nuclear medicine. These drugs may be delivered ready to use, as with 2-deoxy-2-[fluorine-18]fluoro-D-glucose (^18^F-FDG), or they may require preparation in a radiopharmacy laboratory such as for daily used Single-photon-emission-computed-tomography (SPECT) imaging with technetium-99 m preparations. Most of the time, it is a preparation in a multi-dose vial used throughout the day depending on the patients’ arrival. Compliance with hygiene recommendations and Good manufacturing practice (GMP) in a radiopharmacy department is sometimes difficult to achieve because of the challenge in finding a compromise between radiation protection and hygiene or the false security provided by radioactivity that is supposed to be bactericidal (Mattner and Gastmeier 2004; Silberstein 2014). For this purpose and in order to ensure the sterility of a radiopharmaceutical preparation, sterility testing must be performed to guarantee the absence of microbial contamination of the solution. The European Pharmacopoeia recommends that radiopharmaceutical preparations for parenteral administration should comply with the sterility test, despite the particular difficulties in performing this test due to the short half-life of some radionuclides, small batch sizes and radiation risks (Radiopharmaceutical preparation 2023). The latest European Association of Nuclear Medicine (EANM) recommendations on Good Radiopharmacy Practice from Gilllings and al suggests storing samples for sufficient radioactivity decay, and then sent for sterility testing to an external laboratory without an authorization to handle radioactive materials (Gillings et al. 2021). Another way is to perform internal sterility testing, but the lack of equipment to handle these samples and the risk of radiation make this a difficult practice to implement.

Here we present illustrative examples that highlighting the effects of technetium-99 m gamma rays on bacterial strains frequently responsible for injectable solutions contaminations in radiopharmaceutical solutions.

## Materials and methods

### Study design

We followed the recommendations of the European Pharmacopoeia and various authors to determine *s*ensitivity to technetium-99 m (Snowdon 2000; Sterility–European Pharmacopoeia 2023; Dumont et al. 2000; Brown and Baker 1986). Four radioactive solutions of technetium-99 m pertechnetate (^99m^TcO_4_^−^) directly obtained from a ^99^Mo/^99m^Tc generator (1.85, 3.7, 7.4 or 11.1 GBq in 5 mL volume) were inoculated with *Pseudomonas aeruginosa* (*ATCC: 27853*) or *Staphylococcus aureus (ATCC: 25,923)* or *Staphylococcus epidermidis (ATCC: 1228)* to obtain a concentration close to 400 CFU/ml. Samples were then taken after incubation for t = 0 h to 8 h at 25 °C. For each sample, 0.3 ml of solution was plated in triplicate on plates (0.1 ml/agar) and incubated (37 °C/24 h). As reports in the following equation, the average number of viable bacteria was determined (CFU/ml) and standardised against the initial bacterial load inoculated:

$$Mean\, survival \,rate \,at \,t (\%)=100\times \frac{mean \,CFU/mL \,at \,t}{mean \,CFU/mL \,at \,{t}_{0}}$$

As control, the same procedure was performed with non-radioactive technetium-99 control and NaCl solutions.

### Statistical analysis

Analyses were performed using Minitab 19.0 State College, PA: Minitab, Inc (www.minitab.com) and JMP 16.2 statistical software (2010), SAS Institute Inc (www.jmp.com).

Statistical analysis was released in two steps. In the first step, an ANOVA test (1 way) was performed independently for each medium (series: 1.85 GBq, 3.7 GBq,…), in order to identify the series for which the incubation time has an influence on the standardised bacterial load. In the case where the influence of the incubation time is identified as a significant factor in the ANOVA (threshold α = 5%), a Dunnett post-hoc test is performed to compare every mean to the control mean (standardised bacterial load at 0 h in technetium 99).

## Results

First, we showed that the survival rate of the tested microorganisms was not affected by the presence of technetium 99 compared to an isotonic NaCl medium (*p*-value > 0.05).

**Fig. 1:**
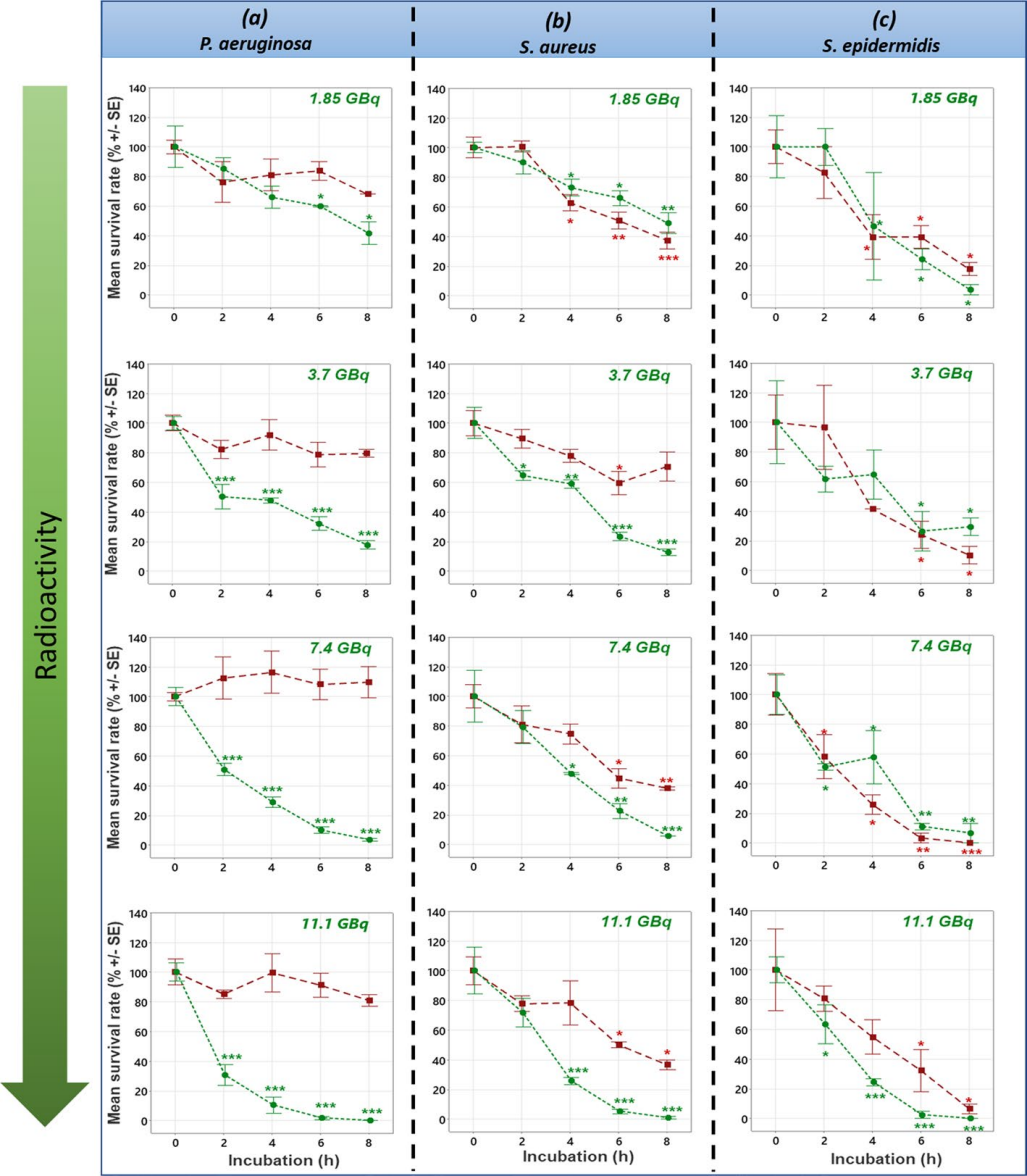
Bacterial survival rate in technetium-99 m radioactive solutions. The survival rate of **a** *Pseudomonas aeruginosa ATCC: 27853* or **b** *Staphylococcus aureus ATCC: 25923)* or **c** *Staphylococcus epidermidis ATCC: 1228* was followed over time (0 to 8 h) in technetium-99 m radioactive solutions at 1.85 to 11.1 GBq (green circle) and non-radioactive technetium-99 solutions (red square). For each incubation time in a radioactive solution, the results are expressed as a percentage of the mean survival rate in relation to the initial bacterial charge load. The asterisk (*) represents the *p*-value of the Dunnett statistical test. One, two and three asterisks means that the *p*-value is respectively less than 0.05, 0.001 and 0.0001

To assess the effects of technetium-99 m gamma rays on bacterial strains, the overtime survival rate of *P. aeruginosa* (*ATCC: 27853*), *S. aureus (ATCC: 25923)* and *S. epidermidis (ATCC: 1228)* incubated in technetium-99 m radiopharmaceutical solutions (1.85 GBq to 11.1 GBq) are reported in Fig. 1*.* Technetium-99 m caused a very important decrease of the number of *P. aeruginosa* CFU in relation to the non-radioactive samples. The stronger the activity applied was, the sooner this effect appeared. For the *S. epidermidis* strain, the results showed a decrease in the number of CFU in both series (radioactive, non-radioactive) with no difference whatever the radioactive activity. Finally with *S. aureus*, the number of CFU decreased in the two series at different times. A more significant decrease was observed in the radioactive series for activities greater than 1.85 GBq. finally.

## Discussion

As reported in Fig. 1a, the average survival rate of *P. aeruginosa* is mainly affected by the radioactivity dose. Indeed, as the dose of radioactivity increases, the red and green curves diverge rapidly with the duration of exposure. On the other hand, *S. epidermidis* (Fig. 1c) seems to be strongly affected by the absence of nutrients in the medium. Indeed, the red and green curves are very close, whatever the dose of radioactivity and the exposure time. Finally, *S. aureus* (Fig. 1b) is an intermediate case where high concentrations of radioelements (11.1 GBq) visibly affect the viability of the microorganism as does the absence of nutrients.

These results highlight that a long delay between the end of the radiopharmaceutical product preparation and the sterility test (to ensure sufficient radioactivity decay of the sample’s before the test) could lead to a false negative result because the analysed product does not correspond to the administered product at t_0_. In order to improve the efficiency and level of sterility controls, it is essential to perform such controls as soon as possible, without waiting for the radioactive decay to avoid invalid quality control measures. Consequently, to ensure patient safety, it is very important to have a complete quality assurance system and to comply with all the relevant hygiene safety measures (strict aseptic techniques, working in a clean room in a class A shielded enclosure, having staff trained and validated by aseptic filling tests, etc.) in order to avoid any contamination during the preparation of a radiopharmaceutical as well as during the dispensing dose for a patient.

Furthermore, from an analytical point of view, the European pharmacopoeia recommends an incubation period for plate cultures to 7 days and not just for 24 h incubation (Fig. 1). This recommendation assumes that, under these conditions, the total bacterial population will be fully capable of recovering from the effects of radiation and lack of nutrients encountered during the product storage period prior to analysis. In our opinion, longer incubation period may eventually allow more effective monitoring of the quality of radiopharmaceutical production practices itself. Despite this, the procedure does not protect patients from microbial contamination, since at best contamination will be detected 7 days later.

## Conclusion

Here we present an example highlighting the action of gamma rays from technetium-99 m on three microorganisms responsible for injectable solutions contaminations. Radioactivity could have an impact on the bacterial viability but this effect was not instantaneous and required high radioactive concentration. Our results therefore run counter to current practice and the latest EANM recommendation of 2021 that radiopharmaceutical preparations should be decayed before sterility testing, particularly when these controls are outsourced to bacteriology laboratories that are not authorized to handle radioactive products^4^. In this case, depending on the nature of the contamination and, of course, the charge of the contamination, such practices can potentially endanger patients. Then, with short plate incubation times (24 h), the patient is exposed to false negative results and with longer plate incubation times (7 days) the patient is exposed to a very late positive response. For patient safety, one solution would be to carry out a microbiological test immediately after production of the radiopharmaceutical, which would have to be stored for 24 h to check its sterility. Unfortunately, this procedure is incompatible with the stability of the technetium-99 m-based radiopharmaceutical.

### Supplementary Information

**Additional file 1.** Raw data of *Pseudomonas aeruginosa* (ATCC: 27853), *Staphylococcus aureus* (ATCC: 25923) and *Staphylococcus epidermidis* (ATCC: 1228) survival rate in technetium-99m radioactive solutions at 1.85 to 11.1 GBq and non-radioactive technetium-99 solutions were reported.

## Data Availability

The raw data is available in the "Additional file 1" section.
